# Materia Medica and Therapeutics

**Published:** 1895-12

**Authors:** 


### BOOK NOTICES.

MATERIA MEDICA AND THERAPEUTICS. A Practical Treatise with Especial Reference to the Clinical Application of Drugs. By John V. Shoemaker, A. M., M. D., LL. D., Professor of Materia Medica, Pharmacology, Therapeutics, and Clinical Medicine, and Clinical Professor of Diseases of the Skin in the Medico-Chirurgical College of Philadelphia; Physician to the Medico-Chirurgical Hospital, Philadelphia, etc., etc. Third edition, thoroughly revised. Reset with new type and printed from new electrotype plates. Royal octavo, pages ix, 1108. Extra cloth, $5.00 net; sheep, $5.75 net. Philadelphia: The F. A. Davis Co., Publishers, 1914 and 1916 Cherry Street.

Twice before has this excellent book been reviewed in the pages of The Homœopathic Physician.

The first edition was brought to the attention of our readers in 1891 in the June number, at page 263. The second edition was noticed in the number for August, 1893, at page 445. In both editions thus noticed it appeared as the second volume of a work of which the first volume was devoted to Pharmacy, Pharmacology, and such therapeutic agents as are not properly classed with drugs. In this third edition it has been published as one volume.

It has been expanded, too, from 680 pages to 1,100. This was necessary by reason of the great increase in the number of new drugs brought before the profession in the last two years, and by reason of incorporating in this volume so many of the chapters that in the first and second editions were kept apart in a separate volume.

Therefore the student loses nothing by having this third edition, since, as above stated, the main points of the former first volume are incorporated with the second, thus making one compact and highly instructive volume.

In the matter of an index it is richly furnished. We find, first, a table of contents by chapters; secondly, an alphabetical table of doses in which the old school dosage of every drug mentioned in the book is given. This occupies seven pages; thirdly, an alphabetical general index of fourteen pages; and fourthly, an alphabetically-arranged clinical index of twenty-three pages. Thus its value is to be rated as in the highest degree a book of reference by reason of these copious indexes.

Like every other materia medica, it gives the name and origin of the drug, its pharmacology, physiology, and therapeutics.

The drugs are arranged in alphabetical order for instant reference, and, as stated in the preface, they include many recent additions, such as acetanilid antipyrin, the creosote derivatives, hydrogen dioxide (though they have seemingly overlooked its concentrated form, the *hydrozone* of Mr. Charles Marchand, so often noticed in this journal), salophen, trional, dermatol, and many others.

"Serum therapy," especially for diphtheria, is also noticed quite elaborately.

It will thus be seen that this work is a veritable encyclopedia and is brought down to the present date.

While no homœopathic physician can make use of it for prescriptions, yet it gives him much valuable information that should be well known by all educated physicians.

